# Indicators for monitoring and evaluating climate change adaptation efforts in South Africa

**DOI:** 10.4102/jamba.v15i1.1426

**Published:** 2023-06-30

**Authors:** Esonasipho Seyisi, Brian Mantlana, Simbarashe Ndhleve

**Affiliations:** 1 Risk and Vulnerability Science Centre, Walter Sisulu University, Mthatha, South Africa; 2 Department of Natural Resources and the Environment, Council for Scientific and Industrial Research, Pretoria, South Africa

**Keywords:** adaptation indicators, climate action, climate policy, metrics, UNFCCC, South Africa

## Abstract

Tracking and reporting on whether countries are implementing climate change adaptation initiatives are increasingly becoming more important, and indicators and metrics for monitoring climate change adaptation have equally become crucial. This study employed systematic literature coupled with expert consultation to identify climate adaptation metrics and indicators using South Africa as a case study. Specifically, this study identifies climate change adaptation indicators and selects indicators suitable for use in South Africa. Thirty-seven indicators of climate change adaptation covering different sectors were identified. Nine were identified as input indicators, eight as process indicators, 12 as output indicators and eight as outcome indicators. Application of the specific measurable achievable realistic and timely (SMART) criterion to the 37 indicators resulted in 18 indicators of climate change adaptation. Following stakeholder consultations, eight indicators were chosen as appropriate for tracking the country’s progress towards climate change adaptation. The indicators developed in this study could contribute to climate adaptation tracking, while offering initial steps towards a set of indicators and their improvement thereof.

**Contribution:**

Insights from this article can provide actionable information for decision-making in climate change adaptation. This is one of the few studies that seek to narrow down relevant and applicable indicators and metrics used by South Africa when reporting climate change adaptation.

## Introduction

At the establishment of the United Nations Framework Convention on Climate Change (UNFCCC), there were concerns that climate change adaptation would sidetrack efforts to mitigate climate change. However, this mindset changed in the mid-2000s (Tompkins et al. 2018), because of the impacts of climate change being observed globally and the willingness of developed countries to assist developing countries with the implementation of measures to adapt to climate change impacts (Bours, McGinn & Pringle 2013). Today, the significance of adaptation as a critical global response to climate change is reflected by the fact that more than 170 countries include adaptation in their nationally determined contributions (NDCs) (Nachmany, Surminski & Byrnes 2019).

Furthermore, the progress report by the UNFCCC secretariat on National Adaptation Plan (NAP) 2020, which serves to guide national climate change actions, indicated that 125 of the 154 developing countries have started actions connected to the process to articulate and implement NAPs (UNFCCC 2020). However, little is known about the extent of how and if these plans have been realised and much less about their impacts. The shift towards a focus on climate change adaptation signals a recognition of the significance of adaptation in responding to climate change (Mohner, Navi & Tawfig 2021; Olazabal & De Gopegui 2021; Runhaar et al. 2018).

It is now well established that the Paris Agreement has enhanced the profile of climate change adaptation in the global response to climate change (Berrang-Ford, Ford & Paterson 2011). However, according to Lesnikowski et al. (2017), for countries to realise the adaptation provisions in the Paris Agreement, there is a need to develop systematic approaches for monitoring progress in adaptation across countries and within the country. Indeed, several authors have called for improvements in climate change adaptation methodological aspects (Adamson, Hannaford & Rohland 2018; Berrang-Ford et al. 2011; Biesbroek, Lourenco & Swart 2014; Hulme 2008; Moser and Boykoff 2013; Ribot 2006; UNESCO 2013). Such a methodological toolbox would serve as a guide to quantify the results of adaptation actions.

To date, there are well-documented challenges facing adaptation methodologies. These include the lack of approved adaptation metrics under the UNFCCC, lack of common definitions for adaptation concepts, vague description of adaptation goals in terms of targets and indicators, and insufficient knowledge of what adaptation looks like (Christiansen, Martinez & Naswa 2018; Dilling et al. 2019; Dow et al. 2013; Ford 2013; Moss et al. 2013; Tompkins et al. 2018).

This article aims to contribute to the development of a methodological toolbox for understanding South Africa’s progress in climate change adaptation. Specifically, we aim to develop indicators to track the progress of climate change adaptation responses using South Africa as a case study. We recognise that numerous definitions of indicators are found in the literature on monitoring and evaluation. In this article, the term ‘indicators’ reflects our view on how this term can be used to examine adaptation progress at different times and geographical scales. As such, in defining indicators, we follow Arnott, Moser and Goodrich (2016) and define an indicator as a quality or trait that suggests (‘indicates’) effectiveness, progress or success.

This study focuses on climate change adaptation indicators because they tend to be more readily understood by people as they are associated with a lot less uncertainty than say climate projections; they do not require specialised expertise to tailor or understand; they are more commonly compatible with the spatial and temporal data needs of decision-makers; they are more relatable and politically acceptable even in polarised political environments; and they can be particularly valuable in overcoming common barriers to adaptation as they include actionable information.

The article starts by describing South Africa’s climate change adaptation policy landscape. This is followed by the description of methodological approach. The final section discusses the results of the study as well as provides the conclusion.

## South African climate change adaptation policy landscape

As a signatory to the UNFCCC, South Africa’s climate change response is strongly influenced by the UNFCCC processes and has kept up to date with developments stemming from the UNFCCC negotiations. The Department of Environmental Affairs serves as the focal point for the government’s response to climate change policy implementation in South Africa (DEA 2017). At the national level, the National Development Plan (NDP) provides a blueprint for South Africa’s transition to an environmentally sustainable, climate resilient, low carbon and just society by 2030. This is supported by the National Climate Change Adaptation Strategy (NCCAS) that acts as a common point of reference for climate change adaptation efforts in the country through the provision of guidelines for adaptation action to all levels of government (DEA 2017).

Just before hosting the 17th Conference of Parties of the UNFCCC in 2011, the South African Cabinet approved the National Climate Change Response Strategy (bb). The two main objectives of the NCCRP are firstly to ‘effectively manage inevitable climate change impacts through interventions that build and sustain South Africa’s social, economic, environmental resilience and emergency response capacity’, and secondly:

… [*T*]o make a fair contribution to the global effort to stabilize GHG concentrations in the atmosphere at a level that avoids dangerous anthropogenic interference with the climate system within a timeframe that enables economic, social and environmental development to proceed in a sustainable manner. (DEA 2011, p. 11)

The NCCAS and NCCRP are the key national policy documents on adaptation. In addition to these, there are various provincial and municipal level adaptation plans, policies and strategies in South Africa (see Appendix 1). Taken together, the development of the existing sub-national government adaptation policy landscape has been prolific. To date, the advancements in the adaptation policy landscape have culminated in South Africa’s Draft Climate Change Bill. These policies require suitable metrics and indicators for the assessment of climate change adaptation at the scale of South Africa.

## Methodological approach

**FIGURE 1:**
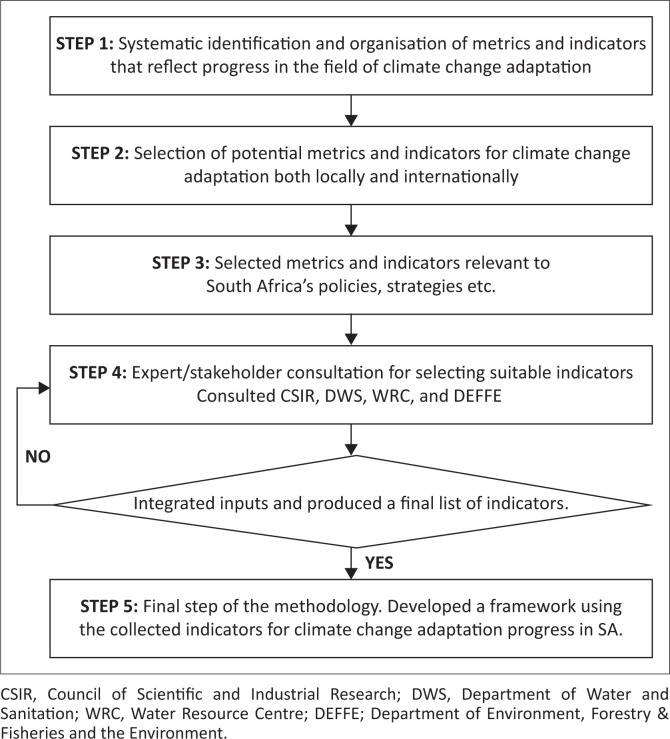
A summary of the methodological steps.

Developing quality climate change adaptation indicators and refining them for local purposes are common practices across countries and regions, and there are numerous studies on the subject (Donatti, Harvey & Hole 2020; Dow et al. 2013; Dudley et al. 2022; Lesnikowski et al. 2017; McCarthy et al. 2012). This section presents the core steps recommended as a guide to the development of quality indicators. These steps are summarised in Figure 1.

Firstly, a systematic literature review approach was used to identify and organise climate change adaptation indicators. Systematic literature reviews have become a common method of analysis in climate change adaptation research (see Araos et al. 2016; Berrang-Ford et al. 2011, 2015; Cherisch & Wright 2019; Carney et al. 2020; Ortega-Cisneros et al. 2021). The systematic literature review was done through the collection of information from three online sources: South African government websites, ScienceDirect and Google Scholar. We searched through these databases using the following words: ‘climate change adaptation’, ‘UNFCCC’, South Africa’s climate change policy landscape’, ‘indicators and metrics’, ‘global climate adaptation’ and ‘progress in climate change adaptation’. To make the literature review search relevant to the national level, we used the following phrases: ‘South Africa’s policy landscape’ and ‘adapting to climate change in SA’.

**FIGURE 2:**
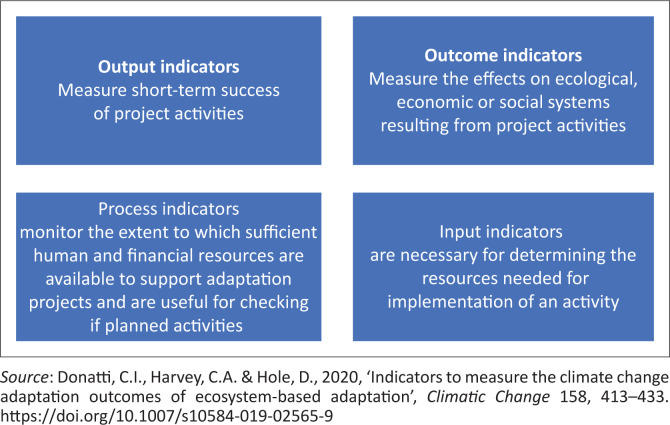
A diagram depicting the definitions of types of indicators.

Indicators that were obtained from the systematic literature review search were then categorised into four types, namely, output, outcome, process and input indicators, following Donatti et al. (2020). Figure 2 gives us a brief explanation of what each indicator type means.

Secondly, the indicators and metrics were then fitted into a specific measurable achievable realistic and timely (SMART) criterion following Nesterova and Van Rooijen (2013) and McCarthy et al. (2012). The SMART criterion has seven criteria and is based on the extent to which an indicator is reliable, is measurable, is complete, is non-redundant, has available data, is familiar and is relevant (see Table 1). In the context of this article, completeness means that the indicator should consider all aspects that affect South Africa’s adaptation goals as outlined in South Africa’s NDCs (DEA 2011, 2017) as well as the adaptation interventions on the NCCAS (DFFE 2020). Familiarity means that the indicator should be easily understood by others and that indicator should be user-friendly, as without proper understanding of what an indicator means it will be difficult to know how to measure it, and the relevance of the indicator is determined by its strong link to the adaptation goals that are in the country’s first NDC.

**TABLE 1:** Criteria for selecting climate change adaptation indicators.

Relevance	The indicator should have a strong link to South Africa’s adaptation goals
Familiarity	The indicators should be easy to understand by the users
Data availability	Data for the indicators should be easily available and be gathered at reasonable costs
Measurability	The identified indicators should be capable of being measured, preferably as objectively as possible
Reliability	The results of the indicators should have a limited degree of uncertainty and margin of error. Factors that increase reliability are good quality of the underlying data, a clear and specific definition of the indicator, and a transparent and direct calculation methodology
Non-redundancy	Indicators within a framework should not measure the same aspect
Completeness	The total set of indicators should consider all aspects that affect the adaptation goals

*Source:* Nesterova, N. & Van Rooijen, T., 2013, *Applied framework for evaluation in CIVITAS PLUS II*, CIVITAS WIKI, Tenerife, Spain.

To complement the literature review, we also presented the identified indicators to climate change adaptation practitioners to solicit their inputs on the identified indicators individually and through focus group discussion. The practitioners were experts in the field of climate change adaptation and government employees.

### Ethical considerations

This article followed all ethical standards for research without direct contact with human or animal subjects.

## Results

A total of 138 publications and online documents were identified through a systematic literature review search by using keywords and phrases. From the list, only 17 publications were relevant to the study published more than a decade ago (2010–2021) (see Table 2).

**TABLE 2:** Articles containing relevant climate change adaptation indicators for the purpose of this study.

Year of publication	Number of articles	Articles (authors)
Before 2016	4	SIDA (2010), Hammill et al. (2014), OECD (2015), Wall and Marzall (2006)
2016	1	Diao (2016)
2018	4	National Treasury (2018), Kirsi et al. (2018), Christiansen et al. (2018), Shah (2018)
2019	3	Leiter et al. (2019), Siders (2019), Magnan and Visiliki (2019)
2020	3	Doubleday et al. (2020), DFFE (2020), Popoola, Shehu & Monde (2020)
2021	2	Vizinho et al. (2021), Flood, Dwyer and Gault (2021)

The identified 17 publications contained 37 relevant indicators that cut across different sectors including the water sector, agricultural sector and energy sector. The 37 indicators were categorised based on the type of indicator they represent. Nine indicators were identified as input indicators, eight indicators were identified as process indicators, 12 indicators were identified as output indicators and eight indicators were identified as outcome indicators.

All the identified 37 indicators were subjected to a two-stage SMART criterion evaluation by an expert or a stakeholder consultation process, firstly as individuals and lastly as a focus group discussion. The results are presented in Table 3.

**TABLE 3:** Identified climate change adaptation indicators.

Indicator type	Description of indicator	Reliable	Measurable	Relevance	Completeness	Non-redundancy	Data availability	Familiarity	Fit to the SMART criterion indicated as %
Input indicators	Number of climate responsive tools developed and tested	✔	✔	✔	✔	✔	✔	✔	100
	Number of vulnerable stakeholders using climate responsive tools to respond to climate variability or climate change	✘	✔	✔	✔	✔	✘	✔	71
	Number of communication tools that incorporate climate change adaptation	✔	✔	✔	✔	✔	✔	✘	90
	Energy storage capacity	✔	✔	✔	✔	✔	✔	✔	100
	Emergency response plans for climate change	✔	✔	✔	✔	✔	✔	✔	100
	Number of financial mechanisms identified to support climate change adaptation	✔	✔	✔	✔	✔	✔	✔	100
Process indicators	Degree of integration of climate change into development planning	✔	✘	✔	✔	✔	✔	✔	90
	Percentage of municipalities with local regulations considering adaptation and vulnerability assessment results	✔	✔	✔	✔	✔	✔	✘	90
	Uptake of measures to reduce air pollution	✔	✔	✘	✘	✔	✔	✔	71
	Targeted groups adopting adaptation responses (including technologies) to ensure a climate resilient society (disaggregated by gender)	✔	✔	✔	✔	✔	✔	✘	90
	Existence of inter-ministerial/intersectoral commissions working on adaptation	✔	✔	✔	✔	✔	✔	✔	100
Outcome indicators	Number of policies, plans or programs introduced or adjusted that mainstream climate risks	✔	✔	✔	✔	✔	✔	✔	100
	Number of policies and coordination mechanisms explicitly addressing climate change and resilience	✔	✔	✔	✔	✘	✘	✔	71
	Percentage of urban households with access to piped water	✔	✔	✘	✘	✔	✔	✔	71
Output	Number of government staff who have received training on adaptation	✘	✔	✔	✔	✔	✔	✔	90
	Number of public awareness campaigns on water efficiency	✔	✔	✘	✘	✔	✔	✔	71
	Number of people supported to cope with the effects of climate change through the availability of a service or facility	✔	✔	✔	✘	✔	✔	✘	71
	Targeted groups adopting adaptation responses (including technologies) to ensure a climate resilient society (disaggregated by gender)	✔	✔	✔	✘	✔	✔	✔	90
	Research in climate change adaptation	✔	✔	✔	✔	✔	✔	✔	100

An indicator is unreliable when there is a lack of information available about that indicator. Collectively, fitness to the SMART criterion for all the indicators ranged from 25% to 100%. Twelve indicators performed well (> 90%) when evaluated using the SMART criterion, while nine indicators were lowly rated (< 50%; see Table 3). Then, indicators were ranked in accordance with the SMART criterion assessment percentage, and only the highly rated factors were selected for consultation. All indicators that scored 70% and above, presented in Table 3, were used for group consultations with climate change experts and stakeholders. However, all 37 indicators were recorded as they could be used for further research in a different context as they did not meet all the requirements for this study (see Appendix 2).

The nine indicators that were found to be unreliable are lacking in terms of how to measure them and there are uncertainties on how information about the indicator will be easily accessible; 25 out of 37 indicators were found to be measurable, and 21 indicators of climate change are relevant. Furthermore, 20 indicators were found to be complete and are part of the adaptation goals outlined in South Africa’s NDC; 29 out of 37 indicators are non-redundant on the list, and two indicators on climate mainstreaming had to be integrated together to avoid the indicator being used twice; 29 indicators passed the data availability criteria, and 22 indicators were identified as familiar.

Table 4 further shows the average percentages of the climate change adaptation indicators that passed the SMART criterion. Amalgamated results for each indicator type and the seven components of the SMART criterion show that input indicators averaged 86%, process indicators averaged 77%, outcome indicators averaged 56% and, lastly, output indicators averaged 64%. Further to that, the analysis shows that when combined, all the indicators scored 60% for both reliability and completeness and a marginally higher percentage (83%) for measurability on indicators. The identified climate change adaptation indicators present shortfalls spread across different components of the SMART criterion.

**TABLE 4:** Comparison of adaptation indicator’s selection criteria.

Indicator type	Reliable (%)	Measurable (%)	Relevance (%)	Completeness (%)	Non-redundancy (%)	Data availability (%)	Familiarity (%)	Fitness to the SMART criterion (%)
Input indicators *N* = 9	78	100	88.9	88.9	88.9	77.8	77.8	85.8
Process indicators *N* = 8	87.5	62.5	75	75	87.5	87.5	62.5	76.7
Outcome indicators *N* = 12	83.3	83.3	25	25	66.7	66.7	41.7	56
Output *N* = 8	50	87.5	50	50	62.5	87.5	62.5	64.3
**Average %**	**74.7**	**83.3**	**59.7**	**59.7**	**76.4**	**79.9**	**61.1**	**-**

Ultimately, a list of 18 climate change adaptation indicators was presented for comments at a focus group session of climate change practitioners. The practitioners decided that the 10 indicators presented should be removed from the final list of indicators because there was not sufficient information to research the indicators further and they were not familiar in the South African context, and thus, only eight indicators were qualified as suitable and applicable to South Africa. After integrating inputs from the climate change practitioners, we developed a set of core indicators that South Africa can use to track its progress on climate change adaptation.

Table 5 shows the final list of eight climate change adaptation indicators. It also reveals that four indicators in the list were input indicators (they can be used for determining the resources needed for the implementation of an activity), two are output indicators (they can measure short-term success of project activities) and two are process indicators (they monitor the extent to which sufficient human and financial resources are available to support adaptation projects and are useful for checking if planned activities did take place). To help with the ease of communication of these indicators, Table 4 also includes an abbreviated version for each indicator as proposed by the stakeholders and experts during the focus group discussion.

**TABLE 5:** Represents the list of climate change adaptation indicators that were developed from the outputs of this study.

#	The list of climate change adaptation indicators collected	Abridged description of the indicator
A	Climate responsive tools (climate investment models, climate risk and vulnerability assessments, and other technical assessments carried out and updated) at different temporal and geo-spatial scales	Climate services
B	Training government officials on climate change adaptation	Capacity development
C	Inter-ministerial/intersectoral (government) structures working on adaptation	Cooperative governance
D	Financial mechanisms to support climate change adaptation, including technical and technological options for adaptation	Financial support
E	Mainstreaming of climate change adaptation into applicable policies, plans and associated processes	Mainstreaming
F	Emergency response plans for climate change	Emergency responses
G	Communication tools that incorporate climate change adaptation	Communication tools
H	Targeted groups adopting adaptation responses (including technologies) to ensure a climate resilient society (disaggregated by gender)	Inclusivity

## Discussion

In this study, we used the SMART criterion following Nesterova and Van Rooijen (2013) and McCarthy et al. (2012) and expert consultation to screen these indicators and came up with eight core indicators that could be used to determine South Africa’s progress on climate change adaptation. All eight indicators are focussing on the ‘what’ aspects of tracking climate change adaptation and are open-ended regarding the ‘how’ aspects of tracking climate change adaptation.

In characterising the set of indicators that were finally developed in this study, we found that some could be regarded as practice-oriented indicators, for example, climate services and emergency responses. Some of the indicators could be regarded as process-oriented indicators, for example, cooperative governance, mainstreaming and inclusivity, while others could be regarded as enablers for climate change adaptation responses, for example, financial support, communication tools and capacity development. In line with these categories, our view is that it is important to indicate the beneficiaries of tracking this information over time. We found that government is the biggest beneficiary of tracking these indicators following the work of Remling and Persson (2014). We also found that government would have the highest responsibility for tracking all these indicators. However, the involvement of non-state actors and communities is critical in tracking five of the eight indicators; these were climate services, emergency responses, inclusivity, communication tools and capacity development.

In further analysing these indicators, we found that all of them can be aggregated to provide a comprehensive understanding over time that they are not time-bound, rather they are continuous; they can be qualitative and quantitative; and they are applicable at all levels of societal organisation (community, local government, provincial government and national level). Moreover, some baseline information on all of them already exists. This would provide a basis upon which improvements can be made over time.

## Conclusion

The findings provide much-needed contextual information about the use of climate change adaptation indicators. The developed indicators could be used as baseline indicators and contribute to a national understanding of climate change readiness and adaptive capacity. This set of indicators can also be readily incorporated as targets in the institutions where the tracking of this information is anchored. They are also applicable to specific initiatives and can be applied across different geographies.

However, we highlight that the identified indicators are particularly relevant at the national level, as it is the national government that creates the enabling conditions for local adaptation, while it ALSO serves as the primary intermediary with the global governance on climate change. Insights from this research can provide actionable information and can form the foundation for more statistically rigorous and generalisable research projects in the future. Future research on this work can include exploring how or whether the identified and other climate change adaptation indicators can facilitate the implementation of adaptation responses. The research design of this article can be improved by thoroughly involving and widening the scope of stakeholders in the selection of indicators. A recognition was made that climate change adaptation cannot await ‘perfect information’ but rather must proceed in the face of uncertainty. Experience and wisdom gained through time, including using such a set of indicators, would be highly valuable.

## Appendix 2: Collected indicators of climate change adaptation

**Table:**

Indicator type	Description of indicator	Reliable	Measurable	Relevance	Completeness	Non-redundancy	Data availability	Familiarity	Fit to the SMART criterion (%)
Input indicator	Number of climate responsive tools developed and tested	✔	✔	✔	✔	✔	✔	✔	100
	Number of vulnerable stakeholders using climate-responsive tools to respond to climate variability or climate change	✘	✔	✔	✔	✔	✘	✔	71
	Number of communication tools that incorporate climate change adaptation	✔	✔	✔	✔	✔	✔	✘	90
	Energy Storage Capacity	✔	✔	✔	✔	✔	✔	✔	100
	Number of cubic metres of water conserved	✘	✔	✔	✔	✔	✘	✔	66
	Number of new major infrastructure projects located in areas at risk	✔	✔	✔	✘	✔	✔	✘	66
	The total sum of investments in programs for the protection of livestock	✔	✔	✘	✔	✘	✔	✔	66
	Emergency response plans for climate change	✔	✔	✔	✔	✔	✔	✔	100
	Number of financial mechanisms identified to support climate change adaptation	✔	✔	✔	✔	✔	✔	✔	100
Process indicators	Degree of integration of climate change into development planning	✔	✘	✔	✔	✔	✔	✔	90
	Percentage of municipalities with local regulations considering adaptation and vulnerability assessment results	✔	✔	✔	✔	✔	✔	✘	90
	Percentage of population living in flood and/or drought-prone areas with access to rainfall forecasts	✔	✘	✘	✔	✔	✘	✔	66
	Uptake of measures to reduce air pollution	✔	✔	✘	✘	✔	✔	✔	71
	Percentage of livestock insured against death because of extreme and slow-onset weather events	✘	✘	✔	✔	✘	✔	✘	44
	Number of new major infrastructure projects located in areas at risk	✔	✔	✘	✘	✘	✔	✘	44
	Targeted groups adopting adaptation responses (including technologies) to ensure a climate resilient society (disaggregated by gender).	✔	✔	✔	✔	✔	✔	✘	90
	Existence of inter-ministerial/ intersectoral commissions working on adaptation	✔	✔	✔	✔	✔	✔	✔	100
	Percentage of poor people in drought-prone areas with access to safe and reliable water	✘	✘	✔	✘	✔	✘	✔	44
Outcome indicators	Number of policies, plans or programs introduced or adjusted that mainstream climate risks	✔	✔	✔	✔	✔	✔	✔	100
	Number of policies and coordination mechanisms explicitly addressing climate change and resilience	✔	✔	✔	✔	✘	✘	✔	71
	Number of properties with retrofitted flood resilience measures; water meters; water efficiency measures; cooling measures	✔	✔	✘	✘	✔	✔	✘	53
	Percentage of treated wastewater	✘	✘	✘	✘	✔	✔	✔	44
	Priority areas for precautionary flood protection	✔	✔	✘	✔	✘	✘	✔	53
	Turnover generated by agricultural cooperatives	✔	✔	✘	✘	✘	✘	✘	25
	Increase in agricultural productivity through irrigation of harvested land	✘	✔	✘	✘	✔	✘	✘	25
	Percentage of households at reduced flood risk because of construction of new or enhanced defences	✔	✔	✘	✘	✘	✔	✘	44
	Percentage of urban households with access to piped water	✔	✔	✘	✘	✔	✔	✔	71
	Percentage of farmland covered by crop insurance	✔	✔	✘	✘	✔	✔	✘	53
	Increase in the percentage of climate resilient crops being used	✔	✘	✔	✘	✔	✔	✘	53
Output	Proportion of forest managers acting on adaptation	✔	✔	✘	✘	✔	✔	✘	44
	Number of methodological guides produced to assess the impacts of extreme weather events on transport systems	✘	✔	✘	✔	✘	✔	✘	52
	Number of government staff that have received training on adaptation	✘	✔	✔	✔	✔	✔	✔	90
	Number of public awareness campaigns on water efficiency	✔	✔	✘	✘	✔	✔	✔	71
	Number of people supported to cope with the effects of climate change through the availability of a service or facility	✔	✔	✔	✘	✔	✔	✘	71
	Conservation of forest genetic resources	✘	✘	✘	✘	✔	✘	✔	25
	Reduction of flood damage and disaster relief costs in cities because of increased standards for flood protection and improved flood emergency preparedness	✘	✔	✘	✔	✘	✔	✘	44
	Targeted groups adopting adaptation responses (including technologies) to ensure a climate resilient society (disaggregated by gender).	✔	✔	✔	✘	✔	✔	✔	90
	Research in climate change adaptation	✔	✔	✔	✔	✔	✔	✔	100
